# A Retrospective Analysis of Seven Bacterial Sexually Transmitted Diseases in the City of Bari: Observations and Future Perspectives

**DOI:** 10.2174/0118715303391063251201145315

**Published:** 2026-06-22

**Authors:** Ciro Gargiulo-Isacco, Stefania Garzone, Del Prete Francesca, Sergey K. Aityan, Vikram D. Sawant, Kieu C.D. Nguyen, Van Hung Pham, Toai Cong Tran, Raffaele Del Prete, Luigi Santacroce

**Affiliations:** 1 Department of Interdisciplinary Medicine, Section of Microbiology and Virology, School of Medicine, University of Bari “Aldo Moro”, Bari 70124, Italy;; 2 Department of Multidisciplinary Engineering, Northeastern University, Oakland Campus, Oakland, CA 94613, USA;; 3 Department of Clinical and Dignostic Microbiology, Viet Nam Research and Development Institute of Clinical Microbiology, Ho Chi Minh City 756000, Vietnam;; 4 Basic Medical Sciences Faculty, Pham Ngoc Thach University of Medicine, Ho Chi Minh City 740500, Vietnam

**Keywords:** Sexually transmitted infections, quantitative polymerase chain reaction, microbiota, *Neisseria gonorrhoeae*, *Ureaplasma urealyticum*, *Ureaplasma parvum*, *Chlamydia trachomatis*, *Mycoplasma hominis*, *Mycoplasma genitalium*, *Trichomonas vaginalis*

## Abstract

**Introduction:**

Sexually Transmitted Infections (STIs) have become a global health problem, causing severe diseases and, in some cases, death. This study aimed to highlight the prevalence and spread of STIs in the Apulian population in the urban area of Bari. This would provide a clearer picture of the onset, persistence, social status, and pathophysiology of STIs, and allow health authorities to identify high-risk groups and personalize prevention and treatment programs.

**Methods:**

This is a retrospective analysis of a study conducted between May 21, 2018, and May 31, 2024, on 6,931 patients. Enrolled patients were of both sexes; swabs, fluid samples, and biopsies were sent to our clinical microbiology laboratory at the Aldo Moro University Hospital in Bari for diagnosis and tested by specific, targeted quantitative Polymerase Chain Reaction (qPCR).

**Results:**

The most frequent infection is caused by *Ureaplasma parvum*, with over 1400 cases. Our data suggest a possible epidemiological correlation between the transmissibility of STIs, cultural and social status, sex, and age. Patients were predominantly women (about 74.9% *versus* 25.1%) and aged between 25 and 50 years. The prevalence of individuals affected by STI-causing microorganisms was significantly higher among younger adults aged 25-55 years, with *Ureaplasma urealyticum*, *Ureaplasma parvum*, and *Mycoplasma hominis* as the major co-infecting agents. In pregnant women, two types of moderate positive correlation were observed: *Mycoplasma hominis* with *Ureaplasma parvum* and *Chlamydia trachomatis*, followed by *Ureaplasma parvum* and *Chlamydia trachomatis*.

**Discussion:**

The study highlights the need for comprehensive care, sensitive to cultural and developmental differences, to address sexuality-related issues among disadvantaged groups, particularly adolescents and young adults. These criteria should be applied nationwide and could serve as a guideline for officials and public health professionals to promote access to sexual health services among these population groups and provide useful information, strengthening preventive measures among younger age groups, and designing sexuality education programs.

**Conclusion:**

By analyzing data (age, gender, race, socioeconomic status, *etc*.) alongside STI diagnoses, we sought to identify patterns and trends in STI prevalence. These data will be crucial for the adoption of evidence-based STI control programs in the Puglia Region. They would eventually help in understanding the specific populations most vulnerable to STIs and the factors contributing to their increased risk.

## 1 INTRODUCTION

Sexually Transmitted Diseases (STDs) are increasing almost everywhere in Italy as well as in the rest of the world, and some sectors of the population are more at risk than others. Social and cultural inequalities can play an important role in health outcomes [1-3]. Several studies conducted around the world have shown that the prevalence of STDs is higher in some groups due to low educational levels, unequal access to healthcare, and low income. Individuals living in poverty are more likely to be diagnosed with STI [4, 5]. In Italy, as in other countries, the least disadvantaged segment of the population, made up of Italians and immigrants, is at higher risk of contracting STDs even without adopting high-risk behaviors [5, 6].

Among these identified factors is the genital, oral, and rectal microbiota, which contribute to the effectiveness of innate and adaptive immunity [7-9]. In this regard, high- throughput molecular sequencing of 16S rRNA gene amplicons has led to a better characterization of vaginal and urogenital microbiota [10, 11]. The results have shown different types of microbiota that diversify across sites, characterized by a scarcity of some genera in favor of others and a diverse set of strict and facultative anaerobes [10, 11]. The microbiota interacts with sexually transmitted pathogens in three ways: (i) different microbes may differentially contribute to the pathophysiology of genital infections and their long-term sequelae [12]; (ii) STI pathogens may influence the composition of the microbiota itself [13]; (iii), the presence of dysbiosis represents the sine qua non condition for the proliferation and long-term settlement of such infections and their corresponding pathogens [14]. This putative biological interaction should then be reflected in a statistical association in both cross-sectional and longitudinal studies. A better understanding of these aspects could lay the foundation for the development of probiotics that will constitute a simpler and more effective preventive approach [13, 14].

This retrospective study includes information on seven sexually transmitted microorganisms, such as *Neisseria gonorrhoeae*, *Ureaplasma urealyticum*, *Ureaplasma parvum, Chlamydia trachomatis, Mycoplasma hominis, Mycoplasma genitalium*, and *Trichomonas vaginalis*. The study aimed to provide a comprehensive analysis of medical records containing patient demographics and information on sexually transmitted diseases, with a sociocultural perspective. Essentially, the analysis aimed, (i) investigating the distribution of infections based on patient age and gender, identifying the most affected age groups and assess any significant differences in infection prevalence between males and females [15]; (ii) analyze infection prevalence and thus, determine which infections are most prevalent in the dataset, and examine how certain infections are distributed across different demographic groups [16]; (iii) identify co-infection patterns, therefore the use of correlation analysis to identify infections that tend to co-occur in patients, highlighting possible co-infection risks, and provide a deeper understanding of how multiple infections are related to one another [17]; (iv) provide practical information and obtain insights that can help health workers diagnose and treat infections based on demographic risk factors and inform public health strategies and preventive measures by identifying high-risk groups and common co-occurrences of infections [18, 19]. Diagnostically, several infections affecting the genitourinary tract are evaluated by screening for signs and manifestations, and if at least three of the following clinical symptoms are present: thin, homogeneous gray discharge, pH, release of a fishy amine-like odor when mixed with 10% potassium hydroxide (positive whiff test), and the presence of clue cells on a wet mount. Regardless of the diagnosis, such infections may occur completely asymptomatic [20, 21].

The study aimed to provide a comprehensive overview of how various STIs may affect different segments of the population, using data visualization and correlation analysis to uncover key trends, infection prevalence, and co-infection patterns, and to clarify the interrelationships between STI pathogens and dysbiosis. These insights can aid in clinical decision-making and targeted healthcare interventions.

## 2 MATERIALS AND METHODS

### 2.1 Study Population

Males and females who attended the Microbiology and Virology Unit in the Department of Interdisciplinary Medicine, Aldo Moro University of Bari, between 2018 and 2024 for routine gynecologic care were included in the study. Informed consent was obtained from all participants. The procedure and data were anonymized and linked to the patient through a numeric code prior to signing the informed consent document. Specifically, patients were referred from the Dermatology Clinic, the Gynecology/Obstetric Clinic, the Infectious Disease Clinic, and other hospital clinics, and some were outpatients. Exclusion criteria were based on the pre-existence of HIV, HCV, HBV, and VRLD infection. A total of 6,931 clinical specimens, comprising urine samples, vaginal swabs, cervical swabs, urethral swabs, and anal swabs, were collected, refrigerated, and transferred within 4 hours to the Hospital Laboratory of Microbiology and Virology Unit.

DNA was isolated from clinical specimens. All samples were collected in sterile screw-cap plastic disposable containers (FL Medical, Torreglia, Italy), while vaginal, endocervical, and urethral specimens were collected using the swabs provided with the commercial kit eNAT™ 2 (Copan Diagnostics, Brescia, Italy). After collection, samples were processed within 72 hours.

### 2.2 Extraction Protocol

Simultaneous analysis for the detection of genomic sequences from several non-viral STI agents (*U. parvum, U. urealyticum, M. hominis, M. genitalium, C. trachomatis, N. gonorrhoeae, T. vaginalis*) was conducted using a multiplex real-time PCR assay, Anyplex™ II STI-7 Detection Kit, with the QIAGEN EZ1 Advanced XL System (Qiagen, Plesmanlaan 125, 1066 CX Amsterdam, The Netherlands). Positivity was defined as test results +++, ++, or +, according to the manufacturer’s manual. The significance of proportion differences was analyzed using the proportion comparison test in MedCalc^®^ software, version 15.11.4 (MedCalc Software, Ostend, Belgium).

Magnetic particle technology, which combines speed and efficiency in nucleic acid purification, involves four steps: lyse, bind, wash, and elute.

1. Cell lysis with Proteinase K: Lysis is performed under highly denaturing conditions and at elevated temperatures. The lysis technique is performed by enzymatic digestion with Proteinase K and lysis buffer to digest viral envelope proteins and inactivate nucleases.
2. Magnetic particle binding: Binding buffer is added to the lysed samples to adjust binding conditions. Lysates are thoroughly mixed with magnetic beads to allow optimal adsorption of viral nucleic acids onto the silica surface. Salt concentration and pH conditions ensure that proteins and other contaminants, which can inhibit PCR and other downstream enzymatic reactions, do not bind to the magnetic particles.
3. Washing of bound nucleic acids: While viral nucleic acids remain bound to the magnetic particles, contaminants are efficiently washed away during a 3-step washing sequence, followed by a final rinse. The plates were loaded into the Bio-Rad CFX96TM Real-Time thermal cycler according to Seegene guidelines. The CFX96TM Real-Time PCR System thermal cycler (Bio-Rad) is connected to a PC on which the CFX96™ Real-Time PCR System programs and the melting curve analysis program (Seegene Viewer) are loaded.

The program for configuring the analysis procedure on the CFX96™ Real-Time PCR System for the detection of 7 types of STI and the internal control IC, can be divided into the following three steps:

1. Setting the protocol;
2. Setting the plate, with 15 μl, PCR Mastermix, 5 μl Sample nucleic acids, 20 μl Total reaction volume;
3. running the analytical session.

### 2.3 Correlation Heat Map for Infections in all Patients and Pregnant Women

The heatmap visualized the correlation between various bacteria in the dataset. The main objective was to determine whether certain infections tend to co-occur in patients (positive correlation) or whether the presence of one infection is associated with the absence of another (negative correlation). The correlation heatmap provided a quick overview of these relationships using color coding (Green for positive correlations, Pink for negative correlations). Numbers in each cell represented the correlation values; values closer to 1 indicate a strong positive correlation (infections occur together more frequently); values closer to -1 indicate a strong negative correlation (infections rarely occur together); values near 0 indicate little or no correlation between the infections.

### 2.4 Statistical Analysis: Mean Age of the Infected Patients

All age estimates were rounded to whole numbers with a 95% confidence level, along with the average (mean) age of male and female patients by infection type. Two-sample t-tests were performed on the mean age for male and female patients for each infection type. We used a significance level α = 0.05; if the *p*-value was less than or equal to α (*e.g*., *p* ≤ 0.05), the null hypothesis was rejected, indicating significance. Results are presented in Tables **1** and **2**.

## 3 RESULTS

### 3.1 Exploratory Data Analysis

#### 3.1.1 Distribution by Age, Gender, and Number of Cases for Each Type of Bacterial Infection

The prevalence of each type of infection, the number of patients, and the percentage of patients by gender are presented as follows. The gender distribution among patients in the dataset provides a clear, proportional representation of how the patient population is divided between males and females. The patient population was predominantly female, accounting for approximately 74.9% of the total patients. Men accounted for a smaller proportion of the dataset, approximately 24.1% (Fig. **1**). The Age distribution of the patients in the STI7 dataset showed that the median age was around 40/45, the center of the dataset, meaning that half of the patients were younger than this age and half were older (Figs. **2a** and **b**). The distribution of patients by infection type is shown in Fig. (**3**). The most common infection among the total was *Ureaplasma parvum*, with almost 23%, followed by *Ureaplasma urealyticum* and *Mycoplasma hominis*, at 6% and 8%, respectively. The distribution of patients by gender showed that *Ureaplasma parvum* was the highest infection among females and males (23%, 17%), followed by *Chlamydia trachomatis* and *Ureaplasma urealyticum* (Fig. **4**); distribution among the female group, pregnant (P yellow) and non-pregnant (NP green) showed that *Ureaplasma parvum* was the highest infection for both (NP23%, P12%), followed by *Chlamydia trachomatis* (NP14%, P8%) and *Ureaplasma urealyticum* (NP12%, 5%) (Fig. **5**).

#### 3.1.2 *Chlamydia Trachomatis* Infection

In the study, the most affected population groups were young adult women and men between 25/45. The peak was between the ages of 25 and 30 for women, with a respective percentage of 37% and 33% (SD 8; CI 2) (α = 0.05); while for men, it was between the ages of 25 and 35, with a respective percentage of 25% and 24% (SD 9; CI 2) (α = 0.05) (Figs. **6a** and **b**).

#### 3.1.3 *Neisseria Gonorrhoeae* Infection

In the study, the most affected population groups were young adult women and men between 20/45. The peak was between the ages of 25 and 30 for women (SD 6; CI 5), with a respective percentage of 29% and 43% (α = 0.05); while for men, it was between the ages of 30 and 35, with a respective percentage of 29% and 25% (SD 9; CI 2) (no significant) (Figs. **7a** and **b**).

#### 3.1.4 *Ureaplasma Urealyticum* Infection

In the study, the most affected population groups were young adult women and men. The peak was between the ages of 30 and 35 for women, with a respective percentage of 24% and 18% (SD 9; CI 1) (α = 0.05); while for men, it was between the ages of 35 and 45, with a respective percentage of 26% and 17% (SD 9; CI 2) (α = 0.05) (Figs. **8a** and **b**).

#### 3.1.5 *Ureaplasma Parvum* Infection

In the study, the most affected population groups are young women and young adult males. The age group for women is between 25 and 45, with a peak incidence of 28% and 21% for women aged 30-35, respectively (SD 8; CI 0) (α = 0.05). For men, the incidence is between 25 and 40, with a peak of 24% and 23% for men aged 35-40 (SD 8; CI 2) (α = 0.05) (Figs. **9a** and **b**).

#### 3.1.6 *Mycoplasma Hominis* Infection

In the study, the most affected population groups are young women and young adult males. The age group for women is between 25 and 40, with a peak incidence of 25% and 25% for women aged between 30 and 35, respectively (SD 9; CI 1) (α = 0.05). For men, the incidence is between 25 and 45, with a peak of 26% and 22% for men aged 35-40 (SD 9; CI 2) (α = 0.05) (Figs. **10a** and **b**).

#### 3.1.7 Mycoplasma Genitalium Infection

In the study, the most affected population groups are young women and young adult males. The age group for women is between 25 and 40, with a peak incidence of 33% and 23% for women aged 35-40, respectively (SD 6; CI 2) (α = 0.05). For men, the incidence is between 25 and 45, with a peak incidence of 23% for men aged between 35 and 35 (SD 8; CI 2) (α = 0.05) (Figs. **11a** and **b**).

#### 3.1.8 *Trichomonas Vaginalis* Infection

In the study, the most affected population groups are young women and young adult males. The age group for women is between 30 and 45, with a peak incidence of 26% and 33% for women aged between 30 and 35, respectively (SD 9; CI 2) (no significant). For men, the incidence is between 25 and 40, with peaks of 28% and 19% for men aged 25-35 and 40 (SD 9; CI 2) (no significant) (Figs. **12a** and **b**).

### 3.2 Correlation Analysis for STI-7 Bacteria

For the correlation coefficients between different infections discussed above that co-occurred in the same patient, if the *p*-value was less than or equal to α (*e.g*., *p* ≤ 0.05), the null hypothesis was rejected, suggesting significance. The results are shown in Tables **1** and **2**.

### 3.3 Correlation Heat Map for Infections in all Patients and Pregnant Women

The correlation values were represented by numbers in each cell; values closer to 1 indicate a strong positive correlation (infections occur together more frequently); values closer to -1 indicate a strong negative correlation (infections rarely occur together); values near 0 indicate little or no correlation between the infections. There was a certain correlation between *Mycoplasma hominis* and *Ureaplasma urealyticum* (+0.22), and *Mycoplasma hominis* with *Ureaplasma parvum* (+0.19) (Fig. **13**). The strongest correlation coefficient among infections co-occurring in pregnant women was observed between *Chlamydia trachomatis* and *Ureaplasma parvum* (+0.33), suggesting that these two bacteria may co-occur in pregnant women. Similarly, although less sensitive, *Mycoplasma hominis* showed a slight correlation (+0.17) with *Ureaplasma parvum*, suggesting a coinfection pattern between these two pathogens in pregnancy.

## 4 DISCUSSION

It is known that STIs can play a significant role in causing additional conditions that create a suboptimal microenvironment for microorganisms associated with Bacterial Vaginosis (BV), urogenital candidiasis, and cancer development [17]. There is a critical interaction between dysbiosis and the invasion of these pathogens. The loss of microbial balance allows these pathogens to produce toxic metabolites, which, in turn, alter immune responses, promoting the inflammatory process and disease progression [18, 19].

Epidemiological measurement of infections due to sexually transmitted pathogens, and the reciprocal impact of Sexually Transmitted Diseases (STDs) on the urogenital and intestinal microbiome, highlights the need for therapeutic and preventive approaches tailored to age, gender, and sociocultural context. Therefore, effective management of this condition requires careful education and information about the pathogenic infection, the resulting microbiome imbalances, and the health consequences [18-20]. STIs-associated BV infections can induce pelvic inflammatory disease, which has been linked to an increased risk of *syphilis, gonorrhoea,* and *trichomoniasis*, as well as incurable viral infections such as Herpes Simplex Virus (HSV), Human Immunodeficiency Virus (HIV), and HPV high-risk related carcinomas. High-intensity urinary *Chlamydia trachomatis* and *Ureaplasma parvum* infections can alter the diversity of the cervicovaginal microbiota, causing BV, which is crucial for maintaining reproductive health [19-22].

In this study, we also documented how these types of infections are shifting by age and social cohort among the very young, adults, and the elderly, but not by gender, as women still constitute the majority of the considered pool [21-23]. Therefore, the challenges these changes may pose to population-level infection estimates need to be addressed. Results obtained in pregnant women were also highlighted, showing that some infections may exhibit higher or lower correlations depending on pregnancy status. Infections in pregnant women can have significant impacts on both maternal and neonatal health, increasing the need for strict monitoring and timely treatment of co-infections during pregnancy [24-26]. Pregnant women may be more vulnerable to certain infections due to altered immune responses, underscoring the need to understand these correlations. Failure to account for gender and cohort changes in performance could potentially lead to systematic bias in population-level infectiousness estimates [25-27].

In this study, the total cohort falls within the age range of 25 to 45 years, and a significant portion of the dataset consists of individuals in their mid-20s to mid-40s. The average age of infected patients is approximately 34 years, with a standard deviation of 9 years. The gender distribution analysis showed that a larger percentage of the dataset consisted of female patients (74.9%) than male patients (24.1%) (Figs. **1** and **2**). The findings could suggest two reasons: women are tested more frequently or are more susceptible to certain infections; however, the precise reason would require further investigation into differences in behavior and healthcare-seeking patterns.

The most frequent infection among all patients was *Ureaplasma parvum,* which exceeded other infections by more than 5 times, with over 1400 cases, followed by *Ureaplasma urealyticum* and *Mycoplasma hominis*. Less common infections included *Trichomonas vaginalis*, *Mycoplasma genitalium,* and *Neisseria gonorrhoeae,* with relatively few cases. However, it is generally accepted that *Ureaplasma parvum* infections are common but often asymptomatic, which might explain the higher prevalence. This suggests that although the typical infected patient is in their early thirties, there is a moderate spread of ages around this average, indicating a diverse age range among infected individuals. In fact, approximately 68% of patients fall within the age range of 25 to 42 years, which is within one standard deviation of the mean, reflecting moderate age variability among infected individuals (Fig. **3**).

Based on gender distribution data, the average age of infected male patients was 36 years, with a standard deviation of 9 years; for infected female patients, the average age was 33 years, with a standard deviation of 9 years. Male patients were, on average, 9% older than female patients within the infected population. Both male and female groups exhibit similar levels of age variability, as indicated by their standard deviations (Fig. **4**).

The average age of non-pregnant infected female patients is 36 years, with a standard deviation of 9 years, indicating a relatively broad age distribution among this group. In contrast, the average age of pregnant infected female patients is lower by 5 years, at 31 years, with a standard deviation of 7 years. This suggests that pregnant infected female patients tend to be younger on average and show 2 years less variation in age compared to non-pregnant infected female patients. These differences, with a 5-year gap in mean age, may reflect demographic or social status trends. Women with low economic income have sexual intercourse at a younger age and have lower levels of school education, which may explain why infection rates are higher among young pregnant women than among older non-pregnant women (Fig. **5**).

Interestingly, the analysis of patients infected with *Chlamydia trachomatis* reveals that the average age of male patients is 34±2 years, with a standard deviation of 9 years, while the average age of female patients is 6 years lower, 28±2 years, with a standard deviation of 8 years. We used a 95% confidence level (sampling error of 0.05 or 5%). Age variance, as indicated by standard deviations, shows moderate dispersion from the mean for both sexes, with males having a slightly broader 1-year age distribution than females (Fig. **6**).

Among individuals infected with *Neisseria gonorrhoeae*, the average age of male patients was 34±2 years, with a standard deviation of 9 years. In contrast, female patients had a lower average age of 28±5 years and a standard deviation of 6 years. The confidence intervals calculated at the 95% confidence level were 2 years for male patients and 5 years for female patients. The sizable difference between the confidence intervals for male and female patients infected with *Neisseria gonorrhoeae* is explained by the small number of infected females (7 patients) compared with the relatively high number of infected males (56 patients). The difference in mean age may indicate potential age-related patterns in infection rates, as male patients tend to be older than female patients. Standard deviations indicate slightly greater variation in age among male patients than among female patients, who have a relatively narrower age range in this sample (Fig. **7**).

For patients infected with *Ureaplasma urealyticum*, the mean age of male patients was 37±2 years, with a standard deviation of 9 years, indicating a moderate age distribution within this group. In comparison, female patients had a slightly lower mean age of 34±1 years, with a standard deviation of 9 years. Estimates were made at a 95% confidence level. This 3-year difference suggests that female patients tend to be slightly younger on average than male patients, although both groups exhibit a similar level of age variability. These age distributions may reflect demographic differences in the prevalence of *Ureaplasma urealyticum* infections between the sexes. A relatively low confidence interval for female patients is explained by a relatively large sample size of female patients (276 patients) compared to 113 male patients (Fig. **8**).

Patients in the *Ureaplasma parvum* group have an average age of 38±2 years, with a standard deviation of 8 years. Female patients have a lower average age of 33 ± 0.4 years, with a standard deviation of 8 years. This 5-year difference in average age may indicate that male patients with this infection tend to be somewhat older than female patients. The comparable standard deviations for both genders suggest a similar level of age variability within each group, indicating that age variation among male and female patients is relatively consistent for this infection. A quite low confidence interval for female patients is due to a significant number of female patients (1410) compared with 86 male patients in the sample. The estimates were made with a confidence level of 95% (Fig. **9**).

The *Mycoplasma hominis* group reported an average age of 39±2 years, with a standard deviation of 9 years. In comparison, female patients have a lower average age of around 33 ±1 years, with a similar standard deviation of 9 years. This 6-year difference in average age suggests that male patients with this infection tend to be older than female patients on average. The relatively high standard deviations for both genders indicate a broad age range among male and female patients, with comparable age variability, suggesting that both groups include patients across a wide age range. A quite low confidence interval for the female patients is caused by a relatively high number of female patients, equal to 275, compared to 57 male patients infected with *Mycoplasma hominis*. The estimates were made with a 95% confidence level (Fig. **10**).

In the *Mycoplasma genitalium* group, 58 male and 37 female patients were infected, with an average age of 36±2 years (standard deviation, 8 years). In contrast, female patients had a lower average age of around 29±2 years, with a standard deviation of 6 years. The 7-year difference in average age suggests that male patients with this infection tend to be older on average compared to female patients. The smaller standard deviation for female patients indicates a narrower age range, while the slightly higher standard deviation for male patients reflects greater age variability. The estimates were made with a 95% confidence level (Fig. **11**).

For patients infected with *Trichomonas vaginalis*, the average age of male patients was 36±3 years, with a standard deviation of 9 years. Female patients have a slightly lower average age of around 34±2 years, with the same standard deviation of 9 years. This 2-year difference suggests a relatively close age distribution between male and female patients with this infection, with male patients being only marginally older on average. The similar standard deviations for both genders indicate a comparable range of age variability, reflecting a broad yet consistent age distribution among both male and female patients. The estimates were made with a 95% confidence level (Fig. **12**).

*Ureaplasma urealyticum* and *Mycoplasma hominis* showed a moderate positive correlation (r = 0.22), suggesting that these infections may co-occur. Similarly, *Ureaplasma parvum* and *Mycoplasma hominis* show a slight positive correlation (r = 0.19), suggesting that these infections may co-occur. There is a very slight positive correlation between *Trichomonas vaginalis* and *Mycoplasma hominis* (0.14), indicating a likelihood of co-occurrence. On the contrary, *Ureaplasma parvum* and *Neisseria gonorrhoeae* show a negative correlation (-0.04), indicating that these infections are less likely to co-occur. Many infections, including those caused by *Mycoplasma genitalium* and others, showed near-zero correlations with other infections, suggesting they tend to occur independently (Fig. **13**).

*Ureaplasma parvum* showed a light-to-moderate positive correlation (+0.33) with *Chlamydia trachomatis*, suggesting that these two bacteria often co-occur in pregnant women. A coefficient of +0.33 indicates a light-to-moderate relationship, not a very strong one, but still a noticeable association. Nevertheless, these findings are consistent with other studies, showing that co-infections between these pathogens are quite common. Similarly, with lower sensitivity, Mycoplasma hominis shows a slight correlation (+0.17) with *Ureaplasma parvum*. Of note, some studies indicate that *Mycoplasma hominis* presence can significantly increase the risk of *Ureaplasma parvum* infection, though the relationship is complex and can vary across populations (Fig. **14**) [22].

### 4.1 General Considerations

In general, there was a difference in the mean age between male and female patients infected with the same type of infection. When these differences were statistically significant, they could be explained by physiological factors or differences in behavioral patterns. We tested the difference in the average ages at the significance level α = 0.05; if the *p*-value was less than or equal to α (*e.g*., *p* ≤ 0.05), the null hypothesis was rejected, suggesting significance. The Null hypothesis stated that there is no difference in the average ages of male and female patients infected with the same infection type, and the alternative hypothesis specified that the average ages of male and female patients differ (Table **1**). The differences in age for both male and female patients infected with *Chlamydia trachomatis, Ureaplasma urealyticum*, *Ureaplasma parvum, Mycoplasma hominis*, and *Mycoplasma genitalium* are statistically significant at the α = 0.05 significance level, and thus should be considered different. On the other hand, the age differences between male and female patients infected with *Neisseria gonorrhoeae* and *Trichomonas vaginalis* are statistically insignificant (Table **2**).

Co-infection with *Ureaplasma urealyticum, Ureaplasma parvum*, and *Mycoplasma hominis* complicates disease progression in the genitourinary tract, serving as a primary cause of inflammation and tissue damage, and predisposing both women and men to secondary infections such as HPV [23-26]. This combination has been seen to drive to severe symptoms, an increased risk of neoplastic events, and overlapping complications. Despite these risks, the routine testing and treatment of asymptomatic or symptomatic individuals for *Ureaplasma urealyticum, Ureaplasma parvum,* and *Mycoplasma hominis* are still not a priority [23-26].

This study has the following interesting points. First, most of our participants had either mild symptoms or were asymptomatic. The genus *Ureaplasma* (*urealyticum* and *parvum*) and *Mycoplasma hominis* were the most common in our six-year cohort. In general, the results on the effects of *Mycoplasma hominis* and even higher levels of *Ureaplasma urealyticum/parvum* are rather spurious or often completely incomplete [23-26]. Second, the sample size was quite large (over 6,931), thereby increasing the study's statistical power. Some trends of change emerged, and statistically significant data were observed [24, 25]. Additionally, negative correlations were observed between certain infections, such as *Ureaplasma parvum* and *Neisseria gonorrhoeae*, indicating that these infections rarely co-occur in the same patient. Infections such as *Chlamydia trachomatis* and *Neisseria gonorrhoeae* were more prevalent among males in certain age groups, whereas *Ureaplasma* and *Mycoplasma* infections were more frequent among females. Gender-specific trends highlighted the need for differentiated public health messaging and prevention strategies that address the unique risks faced by both males and females [24, 25]. These findings could be useful for targeted health interventions, awareness campaigns, or further research on the factors that may contribute to the higher prevalence of this infection among males in specific age groups. By using this visual tool, clinicians and researchers can gain insights into co-infection patterns, which can aid in more targeted diagnostic approaches and treatment strategies [27].

Although urogenital cleanliness was assessed according to Italian standards, dysbiosis scores are also required, which may limit our interpretation, especially when compared with other studies [28, 29]. Finally, our analysis was performed using a cross-sectional design, and the causal relationship between suspected urogenital microbiome changes and bacterial colonization was not examined, which may limit the final observations and conclusions [30-33]. In addition, our cross-sectional study may indirectly reflect the conditions of the urogenital microbiome, which is known to be an ecologically dynamic microenvironment not only during the menstrual cycle but also during different phases of aging [30].

This paper certainly builds on and extends our ongoing body of research, which examines not only the critical factors in the transmission and treatment of STI infections but also the economic and social drivers of antibiotic resistance [32]. In fact, antibiotic treatment for STIs is generally effective, but antibiotic resistance is a growing problem. Infections such as chlamydia, gonorrhea, and syphilis, which are normally treatable with antibiotics, are now increasingly being met with resistance, making treatment more difficult [33]. Importantly, current therapeutic cultures surrounding STIs are predominantly influenced by the almost unassailable assumption that a healthy body is free of pathogens, and by fixed definitions that limit the concept of a “pathogen” to something that does not belong to us [33, 34]. Such a scientific approach shifts the emphasis from the infection as a single organism that causes disease to a more complex and panoramic understanding that considers the future of antibiotic use. This contrasts with the definitive approaches to eradicating the pathogen, which rely solely on increasingly sophisticated medicines that tend to exclude patients' subjectivity and context [35].

Ultimately, these results highlight three main points. (1) Among the difficulties related to the prevention of STDs, the socio-cultural aspect also plays a leading role, with information relating to the importance of dysbiosis and sexual behaviors being critical [33, 34]. Low levels of education and limited knowledge about correct sexual behavior and the role of the microbiota in STIs can hinder the effectiveness of prevention and treatment strategies. This requires a level of awareness and preparation that less well-off individuals typically lack [34-36]. In addition, unlike sexually transmitted infections with visible symptoms such as discharge, burning, pain, and genital ulcers, asymptomatic or minimally symptomatic sexually transmitted infections can spread silently, making them difficult to detect and control [37-40]. This raises issues of both preventive and therapeutic intervention and suggests the need for more appropriate and innovative preventive approaches, as well as greater awareness to encourage regular screening, especially in low and medium-risk populations [41-43]. (2) The difficulty in understanding the impact of dysbiosis in urogenital bacterial infection, in bacterial vaginosis, in obstetric complications, but also in silent infections in males, and in the degree of sterility [44-46]. The relationship between dysbiosis, bacterial vaginosis, obstetric complications, urogenital infections, and infertility remains complex and not yet fully understood. Imbalances in the genitourinary tract microflora and their contribution to adverse outcomes require further research [47-49]. (3) Such a scientific approach shifts the emphasis from the infection as a single organism that causes disease to a more complex and panoramic understanding that considers the future of antibiotic use.

This vision is certainly in contrast with a pathogen-resolving approach, based on the use of increasingly sophisticated drugs, and which forgets the patient and his social context [46]. Understanding these mechanisms is essential for developing effective preventive interventions and treatment protocols targeted to each segment of the population, which will certainly have indirect benefits in reducing the risk of preterm birth, infertility, chronic infections, and other associated complications such as cancer [48-51].

## CONCLUSION

The STI7 data analysis provides critical insights into the patterns of sexually transmitted infections across different age groups and genders. The most significant findings include the high prevalence of *Ureaplasma parvum* and *Ureaplasma urealyticum,* which often co-occur with *Mycoplasma hominis*. Young adults, particularly women in their 20s and 30s, are most affected, underscoring the need for targeted interventions, awareness campaigns, and regular screenings in this demographic. However, understanding these patterns in males and pregnant women is crucial for reducing the risk of complications during pregnancy. Overall, this report emphasizes the importance of proactive public health strategies and ongoing research to address the complex nature of STIs and improve patient outcomes.

## Figures and Tables

**Fig. (1):**
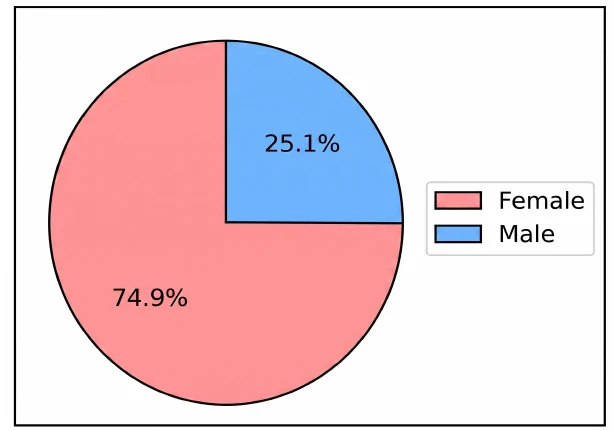
Gender distribution of patients. Male (blue section) represents the percentage of male patients in the dataset. Female (red section) represents the percentage of female patients in the dataset. The patient population is predominantly female, with approximately 74.9% of the total patients being female. Males make up a smaller portion of the dataset, accounting for around 25.1% of the patients. The significant difference in gender distribution might suggest that the study or dataset focuses on conditions more prevalent in females, or that females are more likely to seek treatment in the context of this data.

**Fig. (2):**
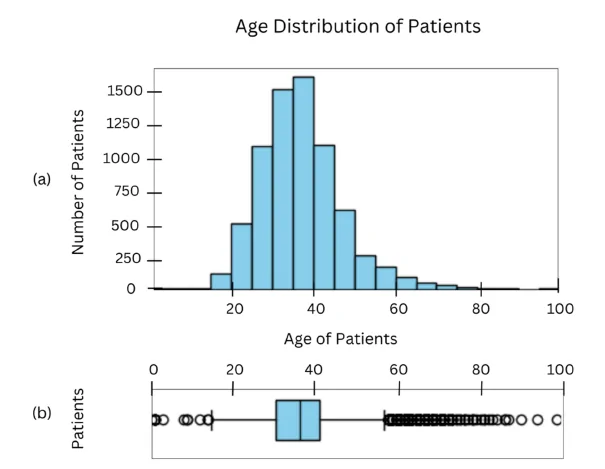
Age distribution of patients, (**a**) histogram, (**b**) boxplot. (**b**) shows the plotbox representation of the same distribution by age as in (**a**). The first Quartile (Q1, 25^th^ percentile) is indicated in the left edge of the box, that shows the age at which 25% of the patient ages fall below this point (lower quartile). Median (Q2, 50^th^ percentile) indicates the line in the middle of the box and represents the median age of the patients. This is the middle value of the dataset, meaning half the patients are younger than this age and half are older. Third Quartile (Q3, 75^th^ percentile) at the right edge of the box indicates the age at which 75% of the patients are younger. This is the upper quartile of the dataset. The maximum refers to the line extending to the right-most point, excluding outliers, and represents the maximum age of patients, excluding unusually high values. Whiskers are the lines that extend from the box to the minimum and maximum values and give the sense of the range of most of the data. They help show where the bulk of the ages lie.

**Fig. (3):**
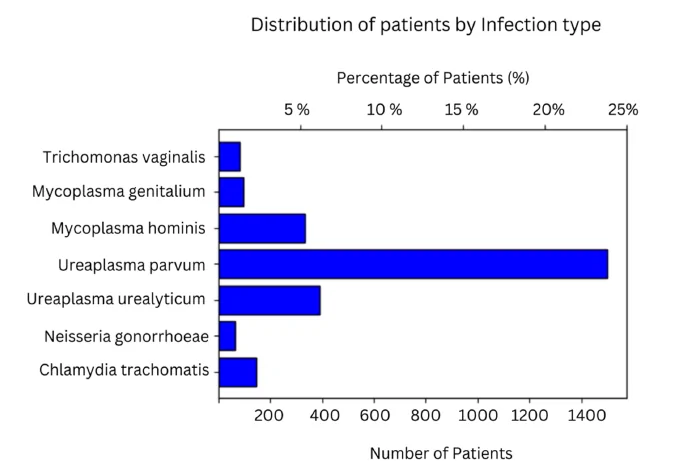
Prevalence of each type of infections, as the number of patients and the percentage of all patients. Horizontal bar chart illustrates the prevalence of different types of infections among patients in the dataset, providing a clear comparison of how common each infection is across the patient population. The various infections are listed in the vertical axis. The lower horizontal axis represents the number of patients diagnosed with each type of infection while the upper horizontal axis represents the same information as the percentage of all patients.

**Fig. (4):**
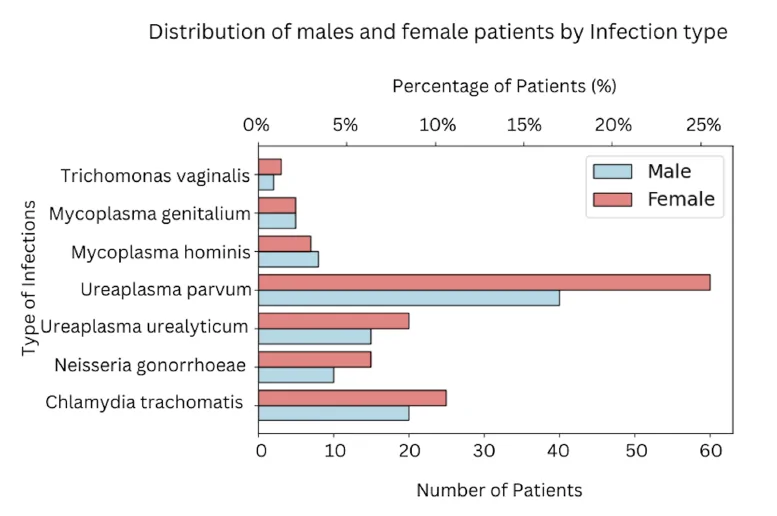
Prevalence of each type of infections, as the number of patients and the percentage of the patients by gender. Urea parvum resulted the highest infectious agent among both male and females patients.

**Fig. (5):**
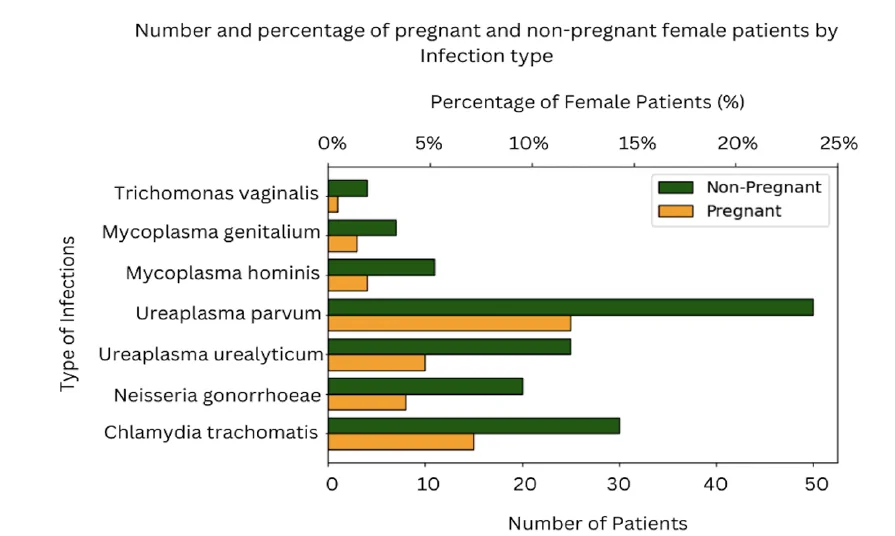
Prevalence of each type of infections, as the number and the percentage of the pregnant and non-pregnant female patients. Urea parvum resulted to be the highest infectious agent among both P and NP women.

**Fig. (6):**
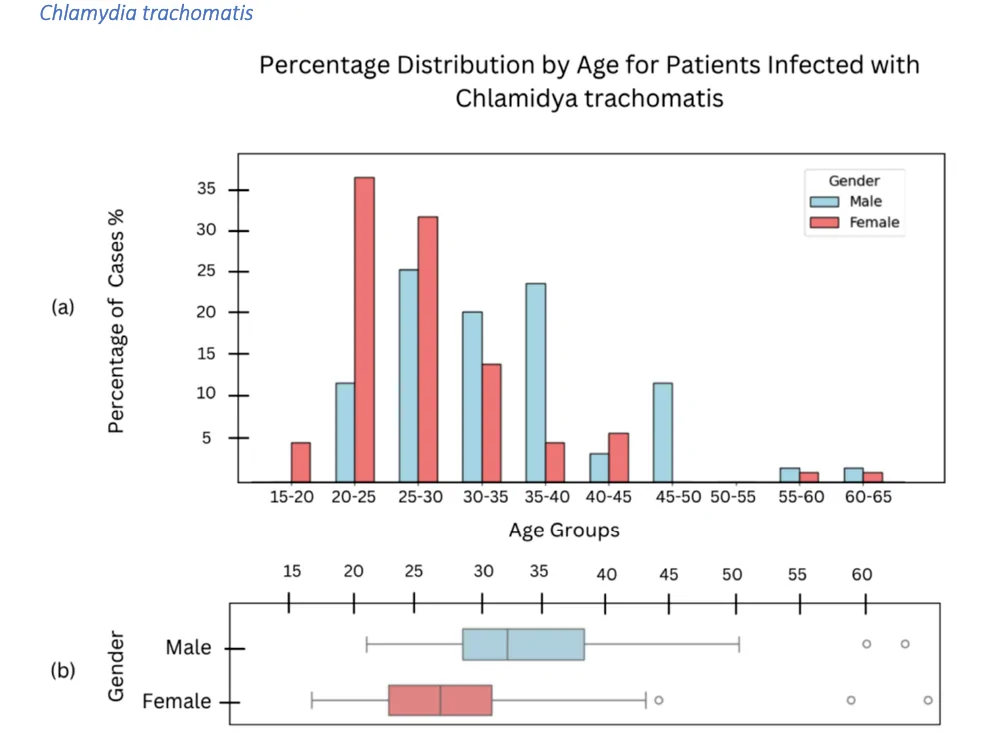
Age distribution for *Chlamydia trachomatis* infection by gender, total number and age (**a** and **b**) percentage of infection cases by gender and by specific age group, *via* boxplot. The histogram and boxplot (**a** and **b**) indicate the prevalence of *Chlamydia trachomatis* infection among different age groups, with a breakdown by gender (males are shown in blue, and females in red). The group most affected by this infection is composed of young women between the ages of 25 and 30. Men are affected to a lesser extent and are in the 35-year age range.

**Fig. (7):**
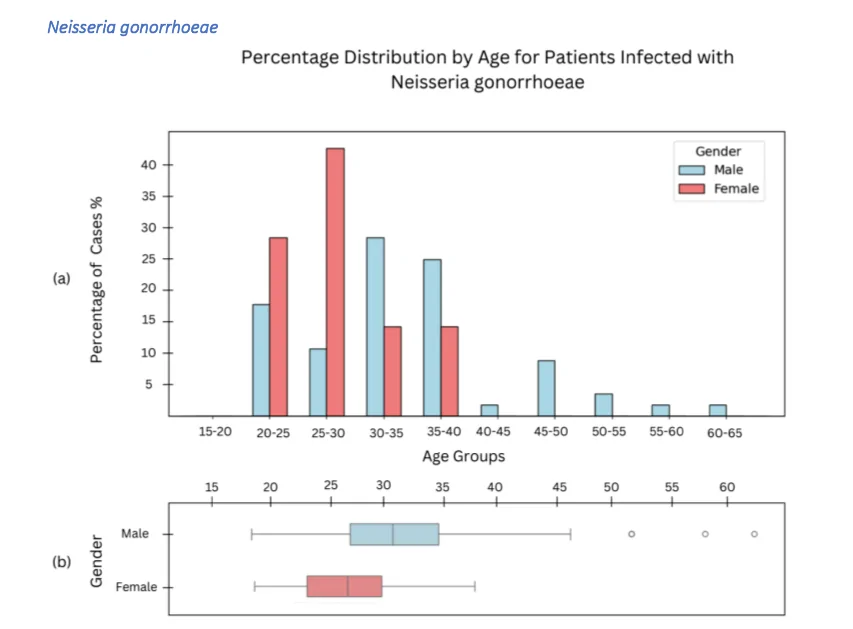
Age distribution for *Neisseria gonorrhoeae* infection by gender, total number and age (**a** and **b**) percentage of infection cases by gender and by specific age group, *via* box plot. The blue bars in the histogram (**a** and **b**) show the percentage of the male patients in the specified age group infected with *Neisseria gonorrhoeae* and the red bars show the percentage of the female patients. The box plot (**b**) visualizes the mean values, percentiles, and outliers in the distribution of the patients infected with *Neisseria gonorrhoeae* across different age groups, with a breakdown by gender. The group most affected by this infection is composed of young women between the ages of 25 and 30. Men are affected to a lesser extent and are in the 30/35-year age range.

**Fig. (8):**
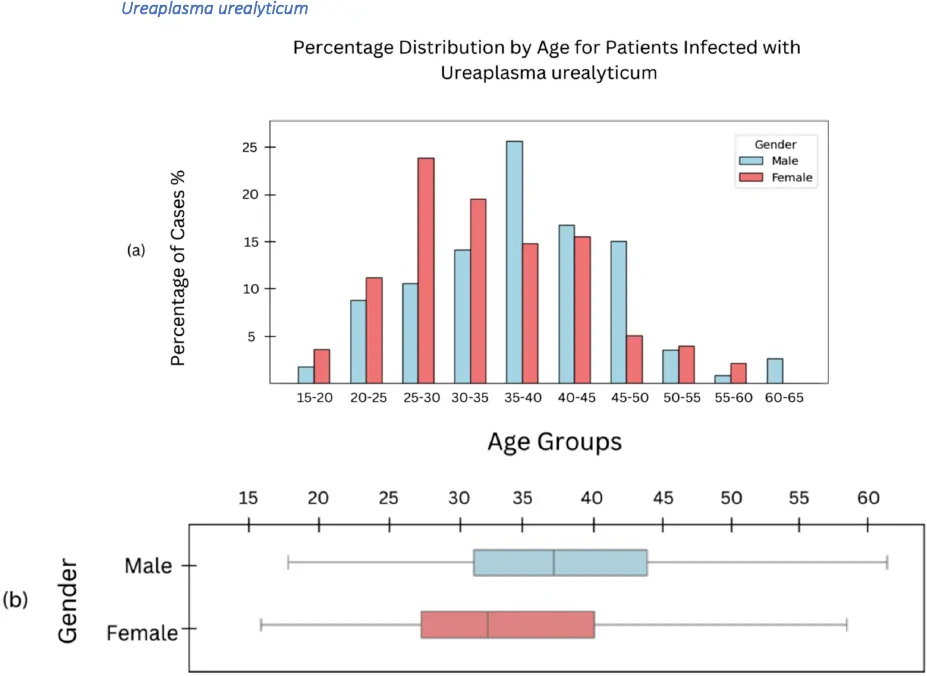
Age distribution for *Ureaplasma urealyticum* infection by gender, total number and age (**a** and **b**) percentage of infection cases by gender and by specific age group, *via* boxplot. The box plot (**b**) visualizes the mean values, percentiles, and outliers in the distribution of the patients infected with *Ureaplasma urealyticum* across different age groups, with a breakdown by gender. The charts help in understanding how the infection impacts different genders across various age ranges. The horizontal axis represents the age groups of patients, ranging from 27/39 for female and 32/43 years old for male. The results showed that this is a prevalent young adult male infection and young female infection between 25 and 45 years old.

**Fig. (9):**
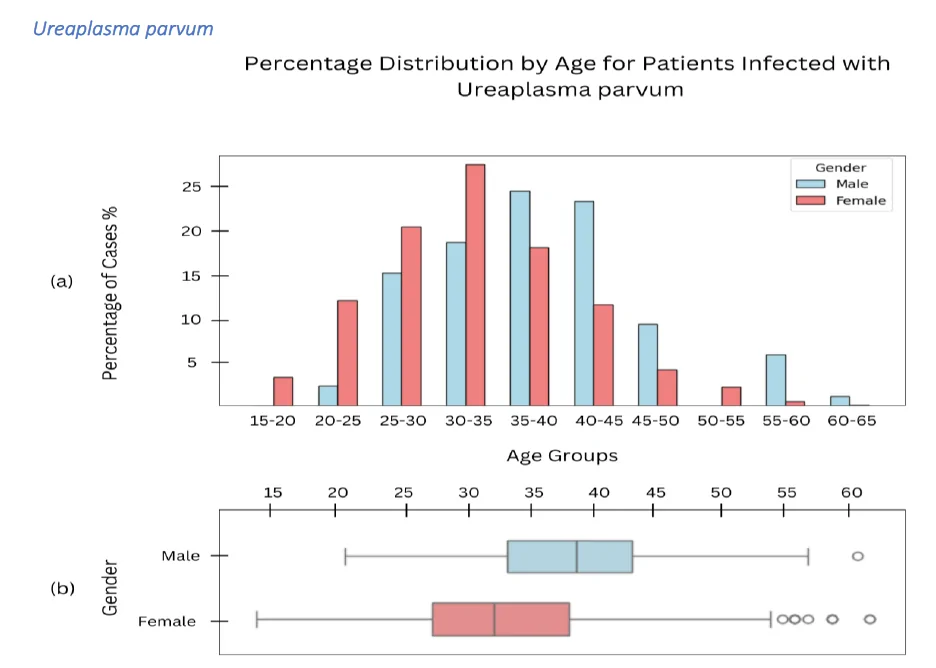
Age distribution for *Ureaplasma parvum* infection by gender, total number and age (**a** and **b**) percentage of infection cases by gender and by specific age group, *via* box plot. The box plot (**b**) visualizes the mean values, percentiles, and outliers in the distribution of the patients infected with *Ureaplasma parvum* across different age groups, with a breakdown by gender. The charts help in understanding how the infection impacts different genders across various age ranges. The horizontal axis represents the age groups of patients, ranging from 26/37 for females to 36/44 years old for males.The results showed that this is a prevalent young adult female infection and young male infection between 25 and 45 years old.

**Fig. (10):**
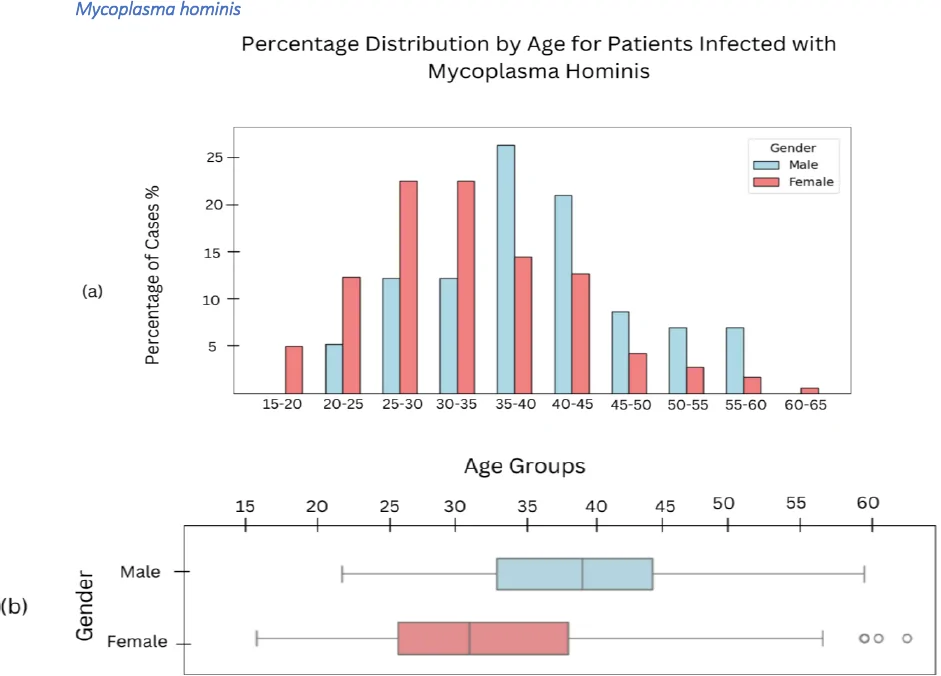
Age distribution for *Mycoplasma hominis* infection by gender, total number and age (**a** and **b**) percentage of infection cases by gender and by specific age group, *via* boxplot. The box plot (**b**) visualizes the mean values, percentiles, and outliers in the distribution of the patients infected with *Mycoplasma hominis* across different age groups, with a breakdown by gender. The charts help in understanding how infection impacts different genders across various age ranges. The horizontal axis represents the age groups of patients, ranging from 25/37 for women, from 33/44 years for men. The results showed that this is a prevalent adult female and male infection between 25 and 45 years old.

**Fig. (11):**
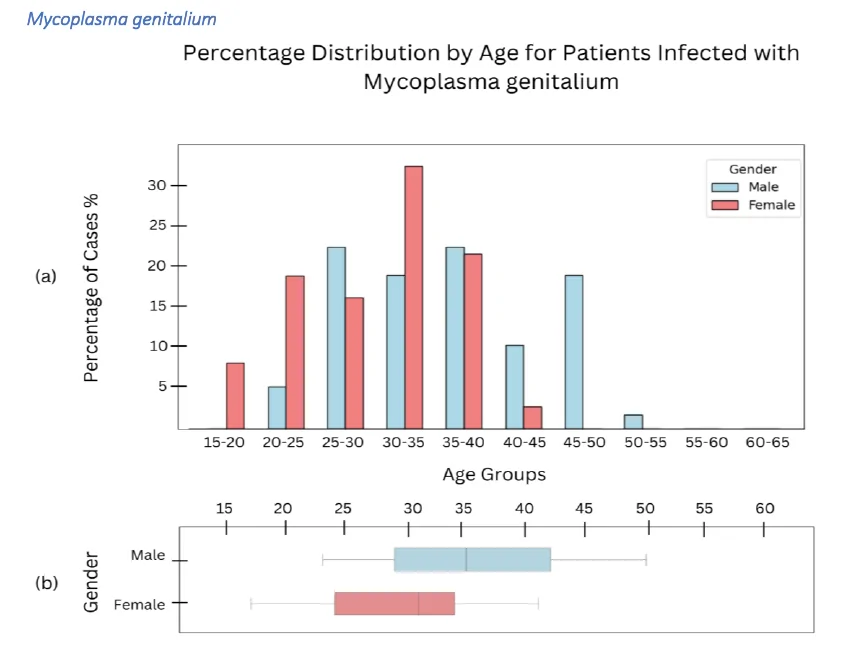
Age distribution for *Mycoplasma genitalium* infection by gender, total number and age (**a** and **b**) percentage of infection cases by gender and by specific age group, *via* boxplot. The charts help in understanding how infection impacts different genders across various age ranges. The box plot (**b**) visualizes the mean values, percentiles, and outliers in the distribution of the patients infected with *Mycoplasma genitalium* across different age groups, with a breakdown by gender. The horizontal axis represents the age groups of patients, ranging from 24/39 for women, from 28/43 years for men.The results showed that this is a prevalent adult female infection between 30 and 40 years old.

**Fig. (12):**
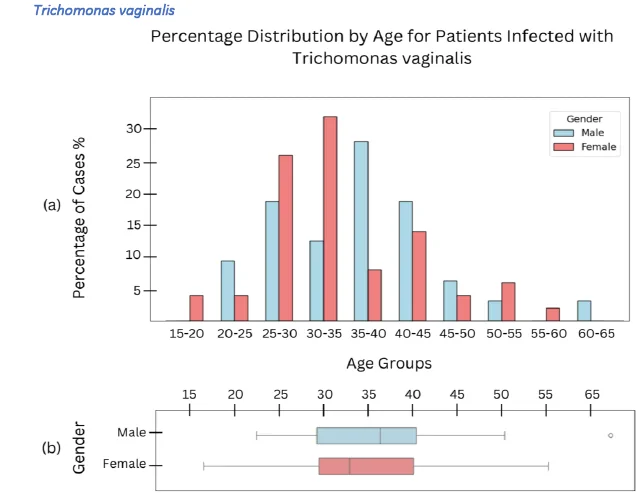
Age distribution for *Trichomonas vaginalis* infection by gender, total number and age (**a** and **b**) percentage of infection cases by gender and by specific age group, *via* boxplot. The charts help in understanding how infection impacts different genders across various age ranges. The box plot (**b**) visualizes the mean values, percentiles, and outliers in the distribution of the patients infected with *Trichomonas vaginalis* across different age groups, with a breakdown by gender. The horizontal axis represents the age groups of patients, ranging from 29/40 for women, from 28/40 years for men. The results showed that this is a prevalent adult female and male infection between 30 and 35 years old.

**Fig. (13):**
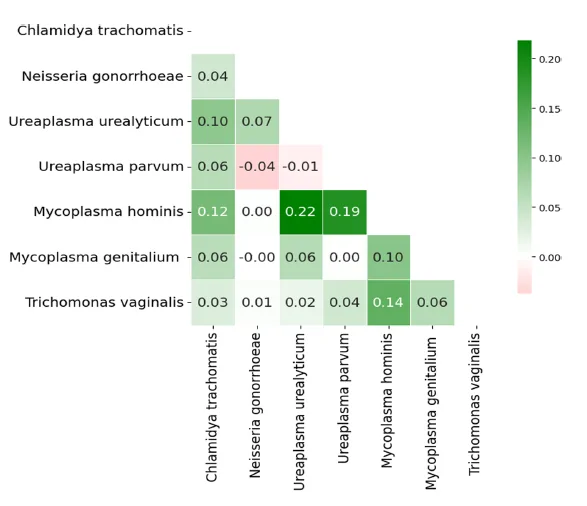
Correlation heatmap for simultaneous infections in all patients. The correlation heatmap provides a quick overview of these relationships using color coding. Green for positive correlations. Pink for negative correlations. Correlation Values: The numbers inside each cell represent the correlation coefficient between two infections. Values closer to 1 show a strong positive correlation (infections occur together more often). Values closer to -1 show a strong negative correlation (infections rarely occur together). Values near 0 show little or no correlation between the infections.

**Fig. (14):**
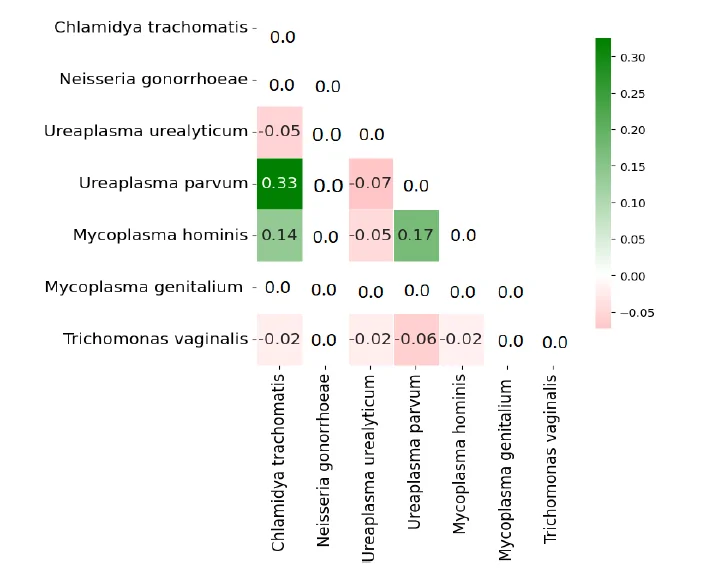
Correlation heatmap for infections in pregnant women. This heatmap visualizes the correlation between different sexually transmitted infections (STIs) pathogens among pregnant women in the dataset. Each value in the heatmap represents the degree of correlation between two infections, with green indicating positive correlations and red indicating negative correlations. The color intensity reflects the strength of the correlation, with darker shades representing stronger correlations. The *Ureaplasma parvum* showed a light moderate positive correlation (+0.33) with *Chlamydia trachomatis*, suggesting that these two bacteria possibly co-occur in pregnant women. This finding aligns with previous research, indicating that co-infections between these pathogens are common. Similarly, with less sensitivity, *Mycoplasma hominis* is slightly correlated (+0.17) with *Ureaplasma parvum*, which may imply a pattern of co-infection between these two as well.

**Table 1:** Summary of mean age of the patients by the infection type.

**Infection Type**	**Male Patients**				**Female Patients**			
	**Mean Age**	**Standard Deviation**	**Number of Patients**	**Confidence Interval (Year)**	**Mean Age**	**Standard Deviation**	**Number of Patients**	**Confidence Interval (Year)**
*Chlamidya trachomatis*	34	9	59	2	28	8	85	2
*Neisseria gonorrhoeae*	34	9	56	2	28	6	7	5
*Ureaplasma urealyticum*	37	9	113	2	34	9	276	1
*Ureaplasma parvum*	38	8	86	2	33	8	1410	0
*Mycoplasma hominis*	39	9	57	2	33	9	275	1
*Mycoplasma genitalium*	36	8	58	2	29	6	37	2
*Trichomonas vaginalis*	36	9	32	3	34	9	50	2

**Table 2:** T-tests on the differences of mean age for the male and female patients by the infection type.

**Infection Type**	**Male Patients**			**Female Patients**			***p-*value**	**Age Difference is Statistically Significant/ Insignificant** **(Significance Level** α **= 0.05)**
	**Mean Age**	**Standard Deviation**	**Number of Patients**	**Mean Age**	**Standard Deviation**	**Number of Patients**		
*Chlamidya trachomatis*	34	9	59	28	8	85	8.1E-05	significant
*Neisseria gonorrhoeae*	34	9	56	28	6	7	6.3E-02	insignificant
*Ureaplasma urealyticum*	37	9	113	34	9	276	4.4E-04	significant
*Ureaplasma parvum*	38	8	86	33	8	1410	2.7E-08	significant
*Mycoplasma hominis*	39	9	57	33	9	275	2.4E-05	significant
*Mycoplasma genitalium*	36	8	58	29	6	37	1.9E-05	significant
*Trichomonas vaginalis*	36	9	32	34	9	50	3.7E-01	insignificant

## Data Availability

The data and supporting information are available in the article.

## LIST OF ABBREVIATIONS

BV: Bacterial Vaginosis
HIV: Human Immunodeficiency Virus
HSV: Herpes Simplex Virus
NP: Non-Pregnant
P: Pregnant
qPCR: Quantitative Polymerase Chain Reaction
STDs: Sexually Transmitted Diseases
STIs: Sexually Transmitted Infections

## STANDARDS OF REPORTING

STROBE guidelines were followed.
